# Alternative boronic acids in the detection of Mycolactone A/B using the thin layer chromatography (f-TLC) method for diagnosis of Buruli ulcer

**DOI:** 10.1186/s12879-023-08426-2

**Published:** 2023-07-27

**Authors:** Gideon A. Akolgo, Benjamin M. Partridge, Timothy D. Craggs, Richard K. Amewu

**Affiliations:** 1 Department of Chemistry, School of Physical and Mathematical Sciences, College of Basic and Applied Sciences, University of Ghana, P.O. Box LG 56, Legon, Accra Ghana; 2 Department of Chemistry, University of Sheffield, Dainton Building, Sheffield, S3 7HF UK

**Keywords:** Buruli ulcer, Arylboronic acid, *Mycobacterium ulcerans*, Buruli ulcer, Mycolactone

## Abstract

**Background:**

*Mycobacterium ulcerans* is the causative agent of Buruli ulcer. The pathology of *M. ulcerans* disease has been attributed to the secretion of a potent macrolide cytotoxin known as mycolactone which plays an important role in the virulence of the disease. Mycolactone is a biomarker for the diagnosis of BU that can be detected using the fluorescent-thin layer chromatography (f-TLC) technique. The technique relies on the chemical derivatization of mycolactone A/B with 2-naphthylboronic acid (BA) which acts as a fluorogenic chemosensor. However, background interferences due to co-extracted human tissue lipids, especially with clinical samples coupled with the subjectivity of the method call for an investigation to find an alternative to BA.

**Methods:**

Twenty-six commercially available arylboronic acids were initially screened as alternatives to BA using the f-TLC experiment. UV–vis measurements were also conducted to determine the absorption maximum spectra of mycolactone A/B and myco-boronic acid adducts followed by an investigation of the fluorescence-enhancing ability of the boronate ester formation between mycolactone A/B and our three most promising boronic acids (BA15, BA18, and BA21). LC–MS technique was employed to confirm the adduct formation between mycolactone and boronic acids. Furthermore, a comparative study was conducted between BA18 and BA using 6 Polymerase Chain Reaction (PCR) confirmed BU patient samples.

**Results:**

Three of the boronic acids (BA15, BA18, and BA21) produced fluorescent band intensities superior to BA. Complexation studies conducted on thin layer chromatography (TLC) using 0.1 M solution of the three boronic acids and various volumes of 10 ng/µL of synthetic mycolactone ranging from 1 µL – 9 µL corresponding to 10 ng – 90 ng gave similar results with myco-BA18 adduct emerging with the most visibly intense fluorescence bands. UV–vis absorption maxima (λ_max_) for the free mycolactone A/B was observed at 362 nm, and the values for the adducts myco-BA15, myco-BA18, and myco-BA21 were at 272 nm, 270 nm, and 286 nm respectively. The comparable experimental λ_max_ of 362 nm for mycolactone A/B to the calculated Woodward-Fieser value of 367 nm for the fatty acid side chain of mycolactone A/B demonstrate that even though 2 cyclic boronates were formed, only the boronate of the southern side chain with the chromophore was excited by irradiation at 365 nm. Fluorescence experiments have demonstrated that coupling BA18 to mycolactone A/B along the 1,3-diols remarkably enhanced the fluorescence intensity at 537 nm. High-Resolution Mass Spectrometer (HR-MS) was used to confirm the formation of the myco-BA15 adduct. Finally, f-TLC analysis of patient samples with BA18 gave improved BA18-adduct intensities compared to the original BA-adduct.

**Conclusion:**

Twenty-six commercially available boronic acids were investigated as alternatives to BA, used in the f-TLC analysis for the diagnosis of BU. Three (3) of them BA15, BA18, and BA21 gave superior fluorescence band intensity profiles. They gave profiles that were easier to interpret after the myco-boronic acid adduct formation and in experiments with clinical samples from patients with BA18 the best. BA18, therefore, has been identified as a potential alternative to BA and could provide a solution to the challenge of background interference of co-extracted human tissue lipids from clinical samples currently associated with the use of BA.

**Supplementary Information:**

The online version contains supplementary material available at 10.1186/s12879-023-08426-2.

## Background

**Fig. 1:**
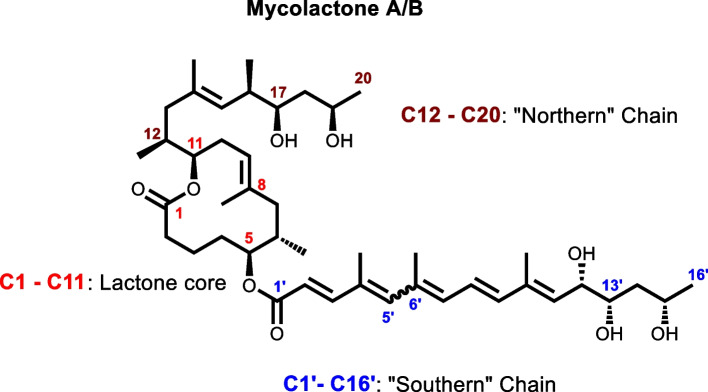
Structure of mycolactone A/B. Mycolactone A/B has a core cyclic lactone ring (C1-C11) and two highly unsaturated acyl side chains produced from polyketides. The longer "Southern" chain is numbered C1′-C16′, and the upper "Northern" chain is made up of C12-C20. Mycolactone exists as a 3:2 ratio of spontaneously generating geometric isomers around the double bond at C4′ C5′ (depicted by the wavy line between C5′ and C6′)

Mycolactone (ML), the first macrolide cytotoxin known to be produced by a human pathogen *Mycobacterium ulcerans* (MU) is the causative agent of Buruli ulcer (BU) [1, 2]. It is the only macrolide identified in the genus Mycobacterium [3]. In 1965, Connor and co-workers hypothesized that *M. ulcerans* was responsible for the production of the diffusible cytotoxin which was subsequently confirmed using guinea pigs. Injection of mycobacterial culture filtrates into the experimental animal resulted in necrosis like those of human infections [4–6]. The suspected molecule was later effectively isolated, purified, and characterized from the acetone-soluble portion of lipid extracts of *M. ulcerans* in 1999 by George et al. The chemical structure was subsequently solved as a polyketide named mycolactone (ML) [7, 8]. ML was detected on thin layer chromatography (TLC) as a light yellow, ultraviolet-active lipid band with a retention factor value of 0.23 in chloroform/methanol/water (90:10:1, vol/vol/vol) [7]. A prominent peak at m/z 765, corresponding to the sodium adduct of mycolactone was observed by mass spectrometry (MS) analysis under electrospray conditions. ML is a hybrid polyketide macrolide with a 12-membered lactone ring at its core, along with a C5-O-linked polyunsaturated acyl side chain numbered C1′-C16′ to the south and a C-linked upper side chain made up of the carbon atoms C12–C20. Different congeners of mycolactone result from differences in the "Southern" chain, while the upper "Northern" chain is invariant. Further research revealed that the mycolactone obtained from *M. ulcerans* was a 3:2 combination of the two stereoisomers known as mycolactones A and B, which form as spontaneous geometric isomers around the double bond at C4′ C5′ (shown by the wavy line between C5′ and C6′) [9, 10] (Fig. 1).

The pathology of *M. ulcerans* disease has been largely attributed to the secretion of this potent cytotoxin, a substance crucial to the pathogenicity of the disease [7]. Tissue necrosis, painlessness, and immunosuppressive properties have all been attributed by various reports to the toxin [11, 12]. Mycolactone A/B diffuses outside the sites of the original infection hence it is an interesting target for detection in circulating blood cells [13].

Since the toxin is only produced by *M. ulcerans*, mycolactone is being investigated as a biomarker for the diagnosis of Buruli ulcer disease. Methods including thin-layer chromatography (TLC) [7, 8], mass spectrometry [14–16], or cytotoxicity assays [15] have been developed to detect and quantify mycolactone in tissue samples towards the diagnosis of the BU disease. Mycolactone has recently been investigated in patient biopsy samples using LC–MS [15, 17] and RNA aptamer binding [18].

**Fig. 2:**
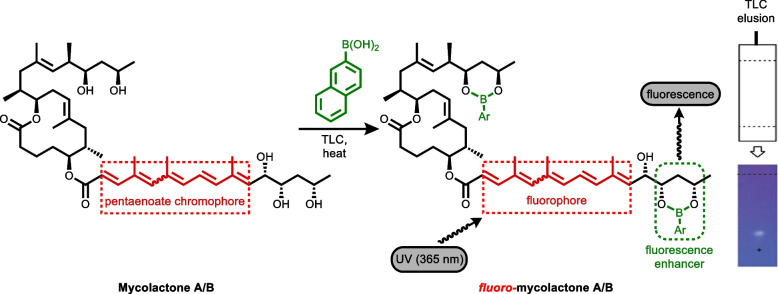
Schematic illustration of fluorescent-TLC detection method showing the derivatization of mycolactone A/B with BA [19]

For diagnosis purposes, a very sensitive, practical, and relatively economical and simple to use fluorescent-thin layer chromatography (f-TLC) method for the detection of the mycolactones produced by the human pathogen, *Mycobacterium ulcerans* was developed by Kishi and colleagues [19]. In the method, mycolactone A/B was chemically derivatized using 2-naphthylboronic acid (BA) as a fluorogenic chemosensor (Fig. 2). The 1,3-diol units on the side chains of mycolactone A/B complex with the boronic acid to generate two 6-membered cyclic boronate rings. When the pentanoate chromophore on the southern side chain was irradiated at 365 nm wavelength, the enhanced fluorescence emission from the C13’, C15’-cyclic boronate was observed at the chromophore emission wavelength of 520 nm [19] (Fig. 2).

Boronic acids have previously been coupled with cis-1,2- and 1,3-diols to generate reversible five- or six-membered cyclic boronate esters [20–23]**.** Fluorescence can be greatly impacted by the formation of boronate esters. The first report on the use of boronic acid as a receptor for saccharide sensing was made in 1992 by Yoon and Czarnik [24]. Since then, numerous biomedical applications have utilised the distinct recognition properties of boronic acids and boronic acid analogues against 1,2- or 1,3-diols [25]. In the area of diagnostic medicine, sensors play crucial roles in their ability to provide quick and quantitative resolution of analytes. Whyte and co-workers showed that boronic acids are reliable sensors for a variety of saccharides, polysaccharides, glycoproteins, glycated proteins, and dopamine [25].

Furthermore, boronic acid sensors have been developed to measure blood glucose levels [26–30], ionic compounds [31–33], water traces in solvents [34, 35], hydrogen peroxide (H_2_O_2_) [36, 37], and catecholamines [38]. Boronic acids have also been employed as biochemical tools for a variety of applications, including tumor cell imaging [39, 40], enzyme inhibitors [41], and cell delivery methods [42]. For these reasons, boron-based fluorescence sensors are crucial in current research.

The detection of mycolactone by coupling to BA has been successfully demonstrated [19, 43–45]. However, concerns such as background interferences from co-extracted human tissue lipids need to be addressed. The search for a more efficient and fluorescent boronic acid as an alternative to the original BA utilized by Kishi and coworkers is required. To find possible alternatives to BA, several commercially available arylboronic acids were screened using f-TLC. The thin layer chromatography (TLC) of the purchased boronic acids was performed and the profile of their fluorescence band intensities was compared to BA.

## Materials and methods

### Reagents and chemicals

Synthetic mycolactone A/B was kindly donated by Prof Kishi Yoshito (Harvard University) through the World Health Organization (WHO), Geneva, Switzerland. All twenty-six structurally diverse arylboronic acids were purchased from Sigma-Aldrich. All reagents were of analytical grade and used without further purification. Solvents are of commercial grade and were used as supplied. Fluorescent dye-free TLC silica gel 60 plates (5721–7) were purchased from EMD chemical Inc and were cut into 5 × 2 cm sizes for the development of the plate. The UV lamp used was a benchtop UV transilluminator (UVLM-28 EL Series UV lamp 365/302 nm (8 Watt) purchased from UVP Inc.

### Spotting

Solutions of 0.1 M of the various structurally diverse arylboronic acids were prepared by the addition of acetone and used according to the previous report of Kishi and co-workers [19]. Standard mycolactone A/B solution for TLC spotting was prepared from a stock solution of 0.1 mg/mL mycolactone in 0.5 mL in an ampoule. A 10 ng/μL standard solution of mycolactone A/B was prepared by taking 0.1 mL of the mycolactone stock solution into a glass vial and adding 0.9 mL ethyl acetate to give the required standard. Optimal visualization of mycolactone A/B was accomplished by spotting the standard on the silica-coated glass TLC plates, eluting with a mixture of chloroform/hexane/methanol (5:4:1; v/v/v) and finally, by slow immersion of the eluted plate in the 0.1 M boronic acid solutions, removal, and then heating the plate to a temperature of ~ 100 °C. The glass side of the plate was then wiped clean with an acetone-soaked tissue paper towel and then viewed by illuminating at ~ 365 nm using a Benchtop UV transilluminator.

### Absorption spectroscopy

The absorption spectra were recorded on a Shimadzu UV-1800 Spectrophotometer. All UV–Vis absorption spectra were obtained using quartz cuvettes with a diameter of 1 cm between 200 and 800 nm.

### Fluorescence spectroscopy

Fluorometric experiments were performed on both a Shimadzu Spectrofluorometer (Model RF-6000) and a Horiba Jobin Yvon (HJY) Fluoromax-3 Spectrofluorometer. Emission spectra were recorded at room temperature with excitation wavelength at 365 nm and slit widths of excitation and emission set at 5 nm and 10 nm respectively. Various dilutions of mycolactone A/B (4 to 32 µg/mL) were prepared from standard solution of mycolactone A/B (0.1 mg/mL in 0.5 mL EtOAc) as shown in the Table 1 below in 4 mL amber vials. They were diluted using cyclohexane to obtain the final concentrations.

**Table 1:** Serial dilutions of mycolactone A/B solutions

No.	0.1 mg/mL mycolactone standard solution (μL)	Diluent (Cyclohexane) (µL)	Final concentration of mycolactone (μg/mL)	Final concentration of mycolactone (µM)
1	**160**	**340**	**32**	**43.1**
2	140	360	28	37.7
3	120	380	24	32.3
4	100	400	20	26.9
5	80	420	16	21.5
6	60	440	12	16.2
7	40	460	8	10.8
8	20	480	4	5.4
9	0	500	0 (Blank)	0

Solutions of boronic acids 2-naphthylboronic acid (BA), [1,1':4',1''-terphenyl]-2-ylboronic acid (BA15), (9,9-Diphenyl-9H-fluoren-4-yl)boronic acid (BA18), and (3-(Naphthalen-1-yl)phenyl)boronic acid (BA21) (0.10 M) in methanol were also prepared. To perform 1 mL reactions, seven samples containing 500 μL of 0.10 M BA18 solution and 500 μL of each dilution of the mycolactone A/B (4 to 32 µg/mL) standard solution in Table 1 were dispensed into 1.5 mL microtubes and mixed well and the fluorescence spectra were recorded after 10 min ( λ_ex_ 365 nm and λ_em_ from 380 to 700 nm).

### General procedure for the preparation of boronate esters from corresponding diols

**Fig. 3:**
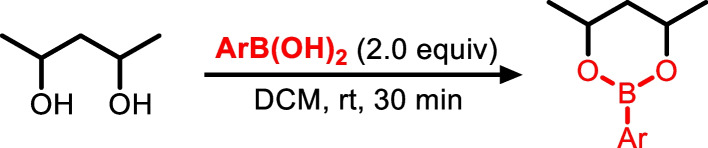
Reaction of 1,3-diol with arylboronic acid to form a 6-membered cyclic boronate ester

To a solution of diol (1 equiv.) in dichloromethane (DCM) (1 M) was added the boronic acid (1 equiv.). The.resultant mixture was stirred for 30 min. The solvent was removed *in vacuo*, and the resultant residue was dried under a high vacuum (0.1 mm Hg) for 1 h. It was then redissolved in cyclohexane and used as a stock solution for mass spectrometry analysis (Fig. 3).

### LC-ESI-Q-ToF analysis

Analyses were carried out using a 1260 Infinity LC (Agilent Technologies), coupled to a Quadrupole-Time of Flight tandem mass spectrometer 6530 Infinity Q-ToF detector (Agilent Technologies) by a Jet Stream ESI interface (Agilent Technologies). The LC conditions were: Zorbax Extend-C18 column (2.1 mm ⨯ 50 mm, 1.8 μm particle size); 0.4 mL/min flow rate; 1 μL injection volume for MS experiments. and 10 μL for MSMS experiments; 40 °C column temperature. Separation was achieved using a gradient of water with 0.1% formic acid (eluent A) and acetonitrile with 0.1% formic acid (eluent B). The elution gradient was programmed as follows: initial conditions 5% B, followed by a linear gradient to 95% B in 15 min. The ESI operating conditions were drying gas (N_2_, purity > 98%): 350 °C and 11 L/min; capillary voltage 4.0 kV; nebulizer gas 40 psig; sheath gas (N2, purity > 98%): 375 °C and 11 L/min. High-resolution MS spectra were acquired in a positive mode in the range 100–2400 m/z.

### Analysis of clinical samples using f-TLC

Clinical samples confirmed by gold standard Polymerase Chain Reaction (PCR) for IS2404 to be either BU or non-BU were analyzed by the f-TLC method using the original boronic acid BA and the most promising boronic acid, BA18 respectively. Both fine-needle aspirates (FNA) and swab samples were analyzed. Standard PCR targeting IS2404 was performed according to the protocol described by Stinear et al. [46].

Mycolactone extraction and detection by fluorescent-thin layer chromatography (f-TLC) technique was done according to the protocol published elsewhere [43]. Ethanol containing the dissolved sample was filtered through a cotton plug into a glass vial. The sample container was further rinsed with 1 mL ethyl acetate, which was added to the glass vial through a cotton plug, and the contents were evaporated to dryness under reduced pressure of about 10–15 mmHg using a rotary evaporator. To separate any contaminating solid from liquid, 100 μL hexane/ether (1:1) solution was added to the glass vial, rinsed, and transferred by microsyringe into a clean glass vial which was air-dried. After evaporation, 50 μL hexane/ether (1:1) was added to the dry sample and 10 μL of the resuspended sample was spotted onto a 3.3 × 6.6 cm fluorescent-dye free TLC plate (TLC Silica gel 60, EMD Millipore, Darmstadt, Germany; Gibbstown, NJ, USA) alongside 50 ng synthetic mycolactone A/B standard in ethyl acetate and a co-spot of 10 μL sample with 50 ng synthetic mycolactone A/B. The plate was developed in chloroform/hexane/methanol (5:4:1; v/v/v) until the leading edge reached the top of the plate, air-dried, and dipped in 0.1 M solution of both BA and (9,9-Diphenyl-9H-fluoren-4-yl)boronic acid (BA18) in acetone, then heated for 60 s at 100 °C on a hot plate. The glass side of the plate was wiped with acetone on a paper towel. The plate was placed on a UV lamp with a 365 nm filter. The fluorescent band at a retention factor of 0.23 from the patient sample was compared to that of the standards to confirm the presence of mycolactone. The f-TLC plates were independently read by two laboratory analysts before reporting the results. The laboratory analysts who worked on both the f-TLC and PCR specimens were blinded to certain relevant clinical information (except for age, and gender) as well as diagnostic test results of each other. This was to ensure that there was no diagnosis bias [43].

## Results

### Fluorescent thin-layer chromatography (f-TLC) analysis

The TLC profiles of twenty-six commercially available aryl boronic acids (Additional document 1, Table S1) were obtained and their fluorescence band intensities profiles were compared to BA. A visual comparison of fluorescent bands on all the TLC profiles indicates that three of the boronic acids (BA15, BA18, and BA21) were visually superior to BA. They gave excellent and highly intense yellow fluorescence bands on a blue background of the TLC plate under 365 nm UV light than BA (Table 2). Following these promising results, the three were selected for further quantitation and spectroscopic studies.

**Table 2:** Structures of the original BA and 3 promising boronic acids with their corresponding TLCs

Entry	Boronic acid	Structure	TLC
BA	2-naphthylboronic acid		
BA15	[1,1':4',1''-terphenyl]-2-ylboronic acid		
BA18	(9,9-Diphenyl-9H-fluoren-4-yl)boronic acid		
BA21	(3-(Naphthalen-1-yl)phenyl)boronic acid		

**Fig. 4:**
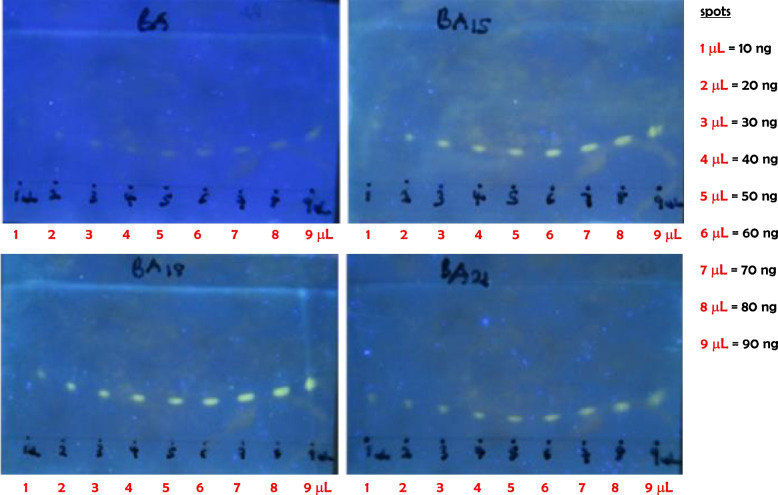
Representative TLC profiles after triplicates experiments for each boronic acid (BA, BA15, BA18 and BA21). Comparison of the original boronic acid (BA) with the three most promising boronic acids (BA15, BA18, and BA21) for the detection of synthetic mycolactone A/B. Various volumes (1 – 9 µL) of the working solution of mycolactone A/B (10 ng/mL) were spotted on a silica gel thin layer chromatography plate, dipped in 0.1 M solution of boronic acids (BA, BA15, BA18, and BA21), eluted with chloroform/hexane/methanol (5:4:1; v/v/v), heated at 100 °C, and illuminated at 365 nm with a benchtop UV transilluminator (UVLM-28 EL Series UV lamp 365/302 nm (8 Watt)

From the prepared working concentration of 10 ng/µL of synthetic mycolactone, various volumes ranging from 1 µL – 9 µL which correspond to 10 ng – 90 ng were spotted on the TLC plate using 1—5 µL calibrated micropipettes (Drummond Scientific). Like the observation above, the three boronic acids gave more intense fluorescent bands compared with BA for all the concentrations. Of the three new ones, BA18 produced the most visibly intense fluorescence bands (Fig. 4).

### UV–vis spectra

**Fig. 5:**
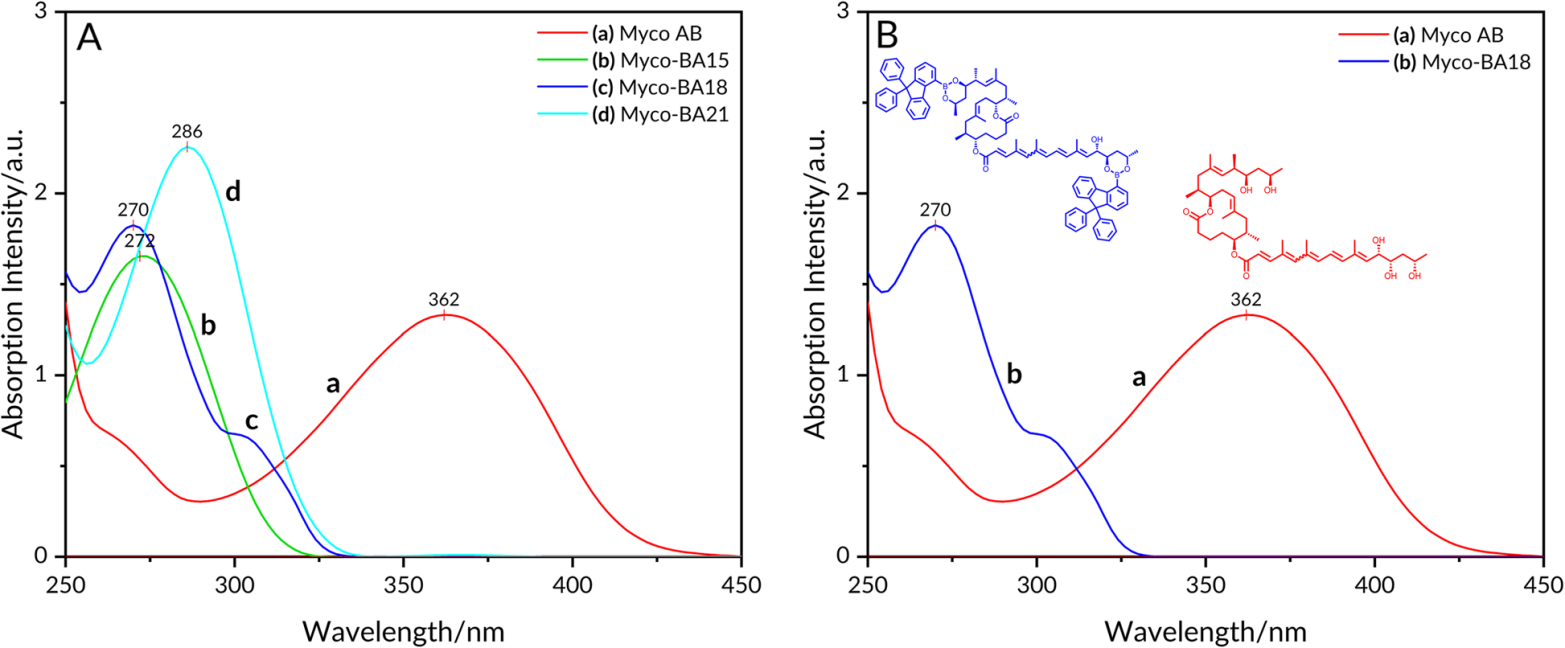
Absorption spectra of mycolactone A/B and myco-boronic acid complexes. **A** (a) mycolactone A/B in MeOH (*λ*_max_ 362 nm); (b) mycolactone A/B – BA15 boronate complex in MeOH ((*λ*_max_ 272 nm); (c) mycolactone A/B – BA18 boronate complex in MeOH ((*λ*_max_ 270 nm); (d) mycolactone A/B – BA21 boronate complex in MeOH (*λ*_max_ 286 nm). **B** Absorption spectra (a) mycolactone A/B in MeOH (*λ*_max_ 362 nm); (b) mycolactone A/B – BA18 boronate complex in MeOH ((*λ*_max_ 270 nm)

Next, we performed UV–vis measurements to determine the absorption maximum spectra of mycolactone A/B and myco-boronic acid adducts. The UV–vis spectra of the free mycolactone A/B and its boronic acids adducts are shown in Fig. 5. The absorption maxima (λ_max_) of the free mycolactone A/B was 362 nm while values of 272 nm, 270 nm, and 286 nm respectively were obtained for myco-BA15, myco-BA18, and myco-BA21.

### Fluorescence spectra of mycolactone A/B– boronic acid complexes

**Fig. 6:**
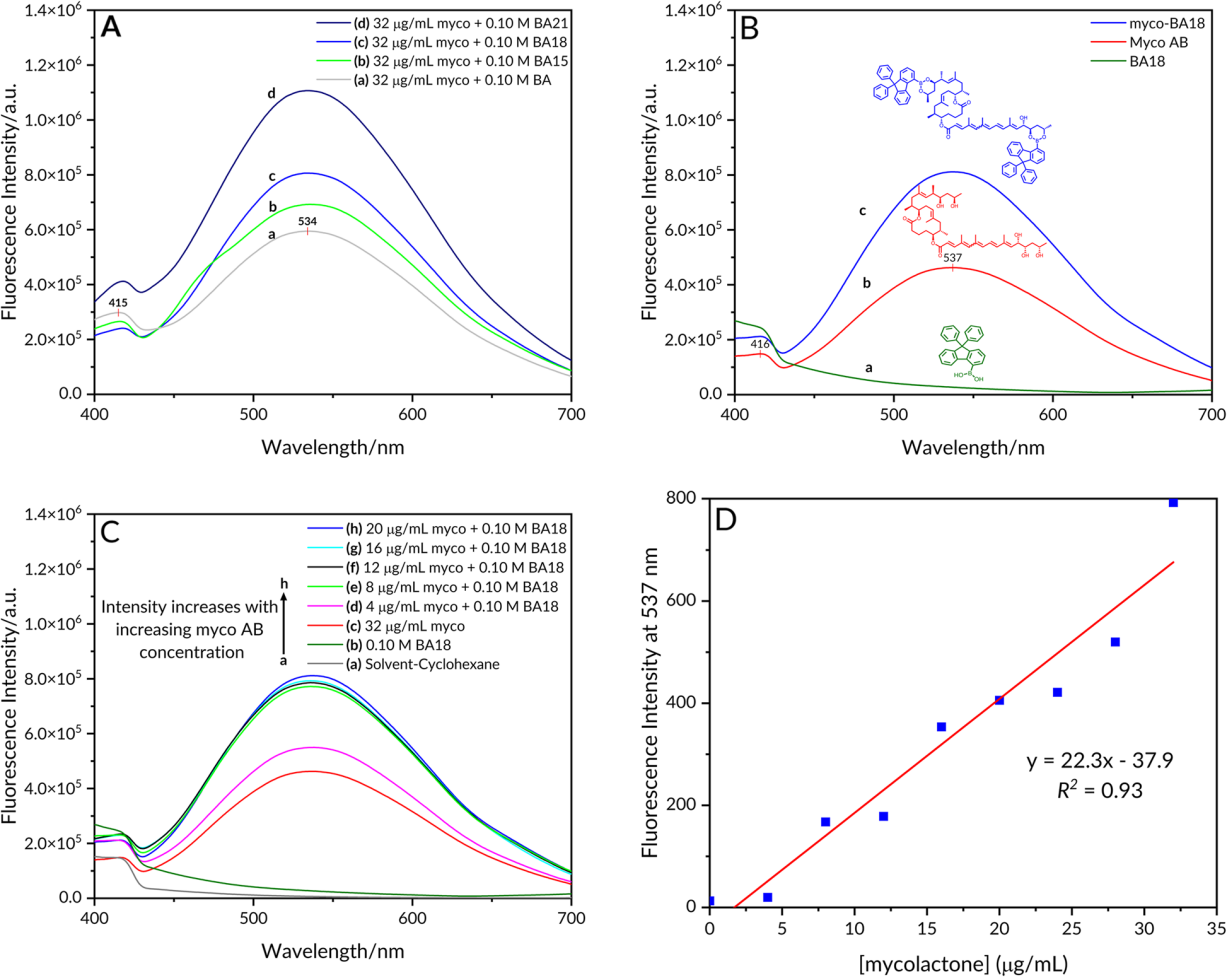
Fluorescence emission spectra in cyclohexane recorded at room temperature with Horiba Jobin Yvon (HJY) Fluoromax-3 spectrofluorimeter. Excitation wavelength 365 nm (excitation slit width 5 nm, emission slit width 10 nm). **A** (a) mycolactone A/B–BA boronate complex; (b) mycolactone A/B–BA15 boronate complex; (c) mycolactone A/B–BA18 boronate complex; (d) mycolactone A/B–BA21 boronate complex; (*λ*_max_ 534 nm); **B** Fluorescence emission spectra (a) BA18; (b) mycolactone A/B in cyclohexane (*λ*_max_ 537 nm); (c) mycolactone A/B–BA18 boronate complex ((*λ*_max_ 270 nm); **C** (a) solvent (cyclohexane); (b) free BA18 (0.10 mol dm^−3^); (c) Free mycolactone (32 µg/mL); (d–h) Mycolactone A/B–boronic acid 18 complexes, BA18 concentration was fixed at 0.10 mol dm^−3^ in the mixtures, while mycolactone concentrations were 4 µg/mL, 8 µg/mL, 12 µg/mL, 16 µg/mL, and 20 µg/mL, respectively, in the mixtures; **D** A plot of fluorescence intensity at 537 nm against various concentrations of mycolactone A/B

In another experiment, we assessed the fluorescence-enhancing ability of the boronate ester formation between mycolactone A/B and our three most promising boronic acids (BA15, BA18, and BA21) (Fig. 6A). When mycolactone A/B was excited at 365 nm in cyclohexane, the pentaenoate gave fluorescence emission at 537 nm. After coupling BA18 to the 1,3-diol, the fluorescence intensity was remarkably enhanced at the same wavelength (Fig. 6B). The fluorescence intensity was also observed to have increased as the concentration of mycolactone increased from 0 to 20 µg/mL Fig. 6C. When the fluorescence intensity at 537 nm was plotted against the concentration of mycolactone, a linear calibration curve with a regression coefficient of 0.93 was obtained (Fig. 6D).

**Fig. 7:**
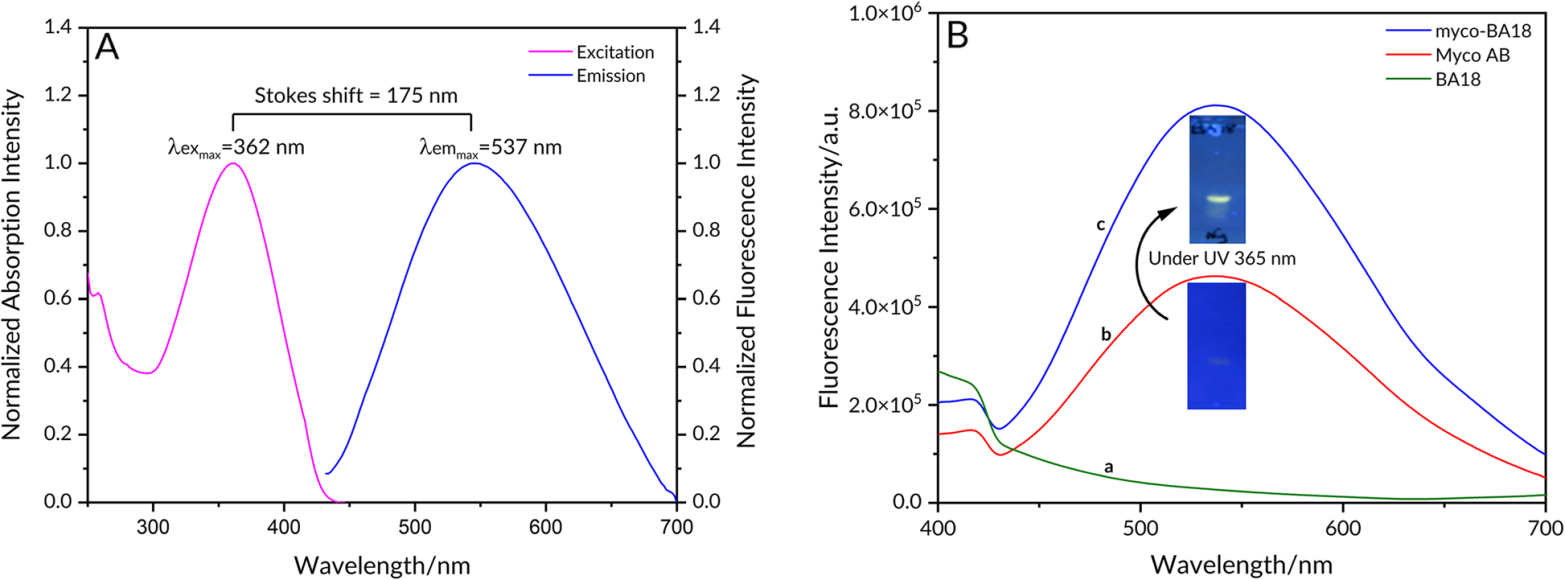
**A** Emission (λ_max_ 537 nm) and its corresponding excitation ( λ_max_ 362 nm) spectra obtained for myco-BA18 boronate complex in cyclohexane and methanol respectively; **B** myco-BA18 boronate complex under 365 nm UV

To examine the correlation between excitation and emission spectral shifts, we plot the excitation peak versus the corresponding emission peak for myco-BA18 boronate complex in cyclohexane and methanol respectively (Fig. 7A). From the plot, we observed an emission peak maximum at a higher wavelength (λex_max_ 537 nm) as compared to the excitation peak maximum (λem_max_ 362 nm). We then determined the Stokes’ shift for the myco-BA18 boronate complex from the excitation and emission spectral peak maxima to be 175 nm. A significant fluorescence enhancement was visually observed on the TLC at 537 nM with (blue) the boronic acid compared to that without (red) using a 365 nM UV lamp (Fig. 7B).

### Mass spectra of mycolactone–boronic acid adducts

**Fig. 8:**
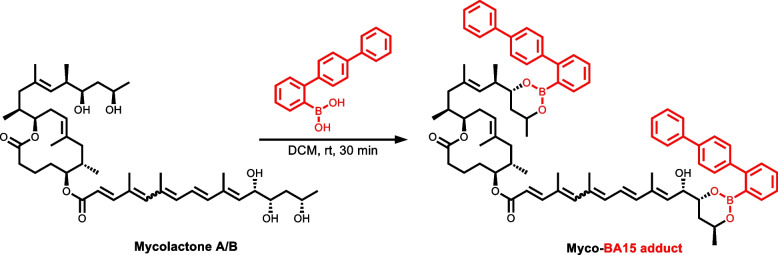
Proposed complexation reaction between boronate 1,3-diols of mycolactone A/B and BA15 to form cyclic boronate adduct

High-resolution mass spectroscopic (MS) analysis was performed to confirm the formation of the mycolactone–BA15 adduct. First, MS analysis of mycolactone A/B was performed, and the chromatogram obtained is shown in (Figure S1). The molecular ion peak was obtained at *m/z* 765.4760 with a formula of C_44_H_70_O_9_Na which corresponds to the sodium adduct of mycolactone A/B. This is consistent with previously published literature mass for the toxin [7]. Then, a chemical reaction according to Fig. 8 was conducted between mycolactone A/B and the B15 after which the mass spectrometer analysis was repeated.

**Fig. 9:**
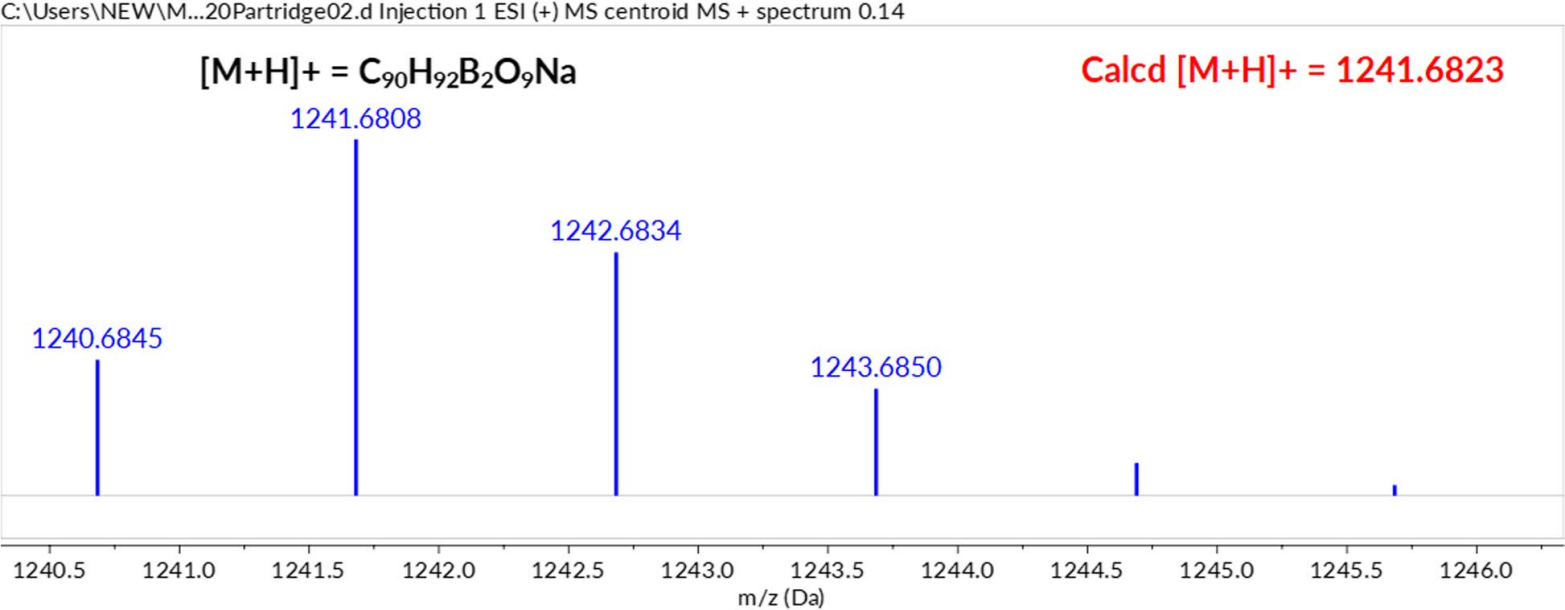
Electrospray Ionization (ESI) mass spectrum of mycolactone A/B–BA15 boronate adduct

The success of the reaction was confirmed by HR-MS analysis. The results showed that the myco-BA15 boronate ester adduct was formed as shown in Fig. 9 below and Additional document 1, Figure S2 A new *m/z* signal observed at 1241.6823 corresponding to [M + Na]^+^ is attributed to the myco-BA15 sodium adduct with elemental formula C_80_H_92_B_2_O_9_Na, confirming the formation of the required adduct. In addition, the MS data strongly corroborated the intensity changes observed in UV–vis and fluorescent experiments.

### Validation on real samples

**Fig. 10:**
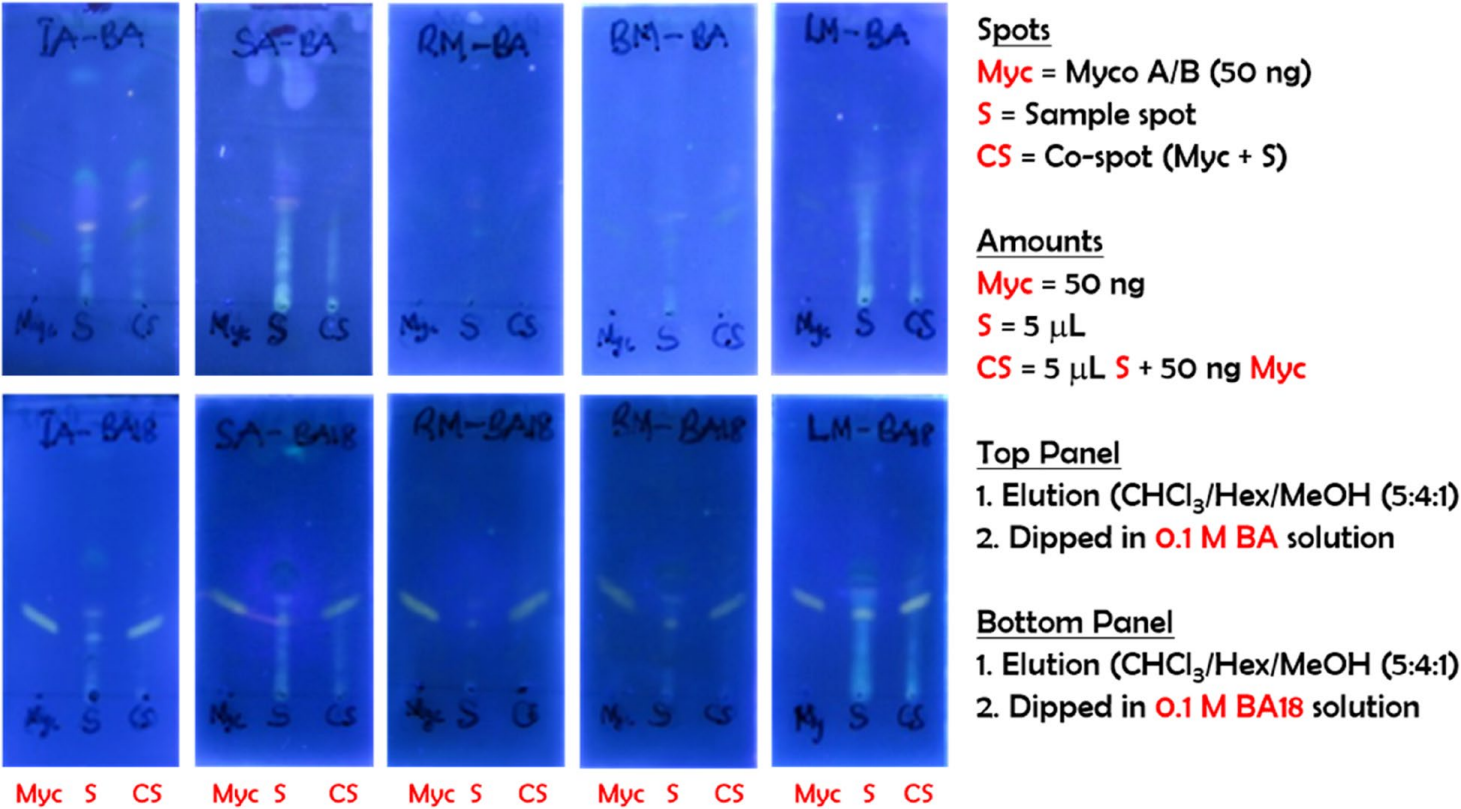
f-TLC plates of clinical samples from 5 different PCR positive confirmed BU cases. Each TLC plate is marked with three spots, Myco, S and CS for synthetic mycolactone A/B, sample and co-spot respectively. The samples were applied at the same concentration and the plates were developed using chloroform/hexane/methanol (5:4:1; v/v/v)

**Fig. 11:**
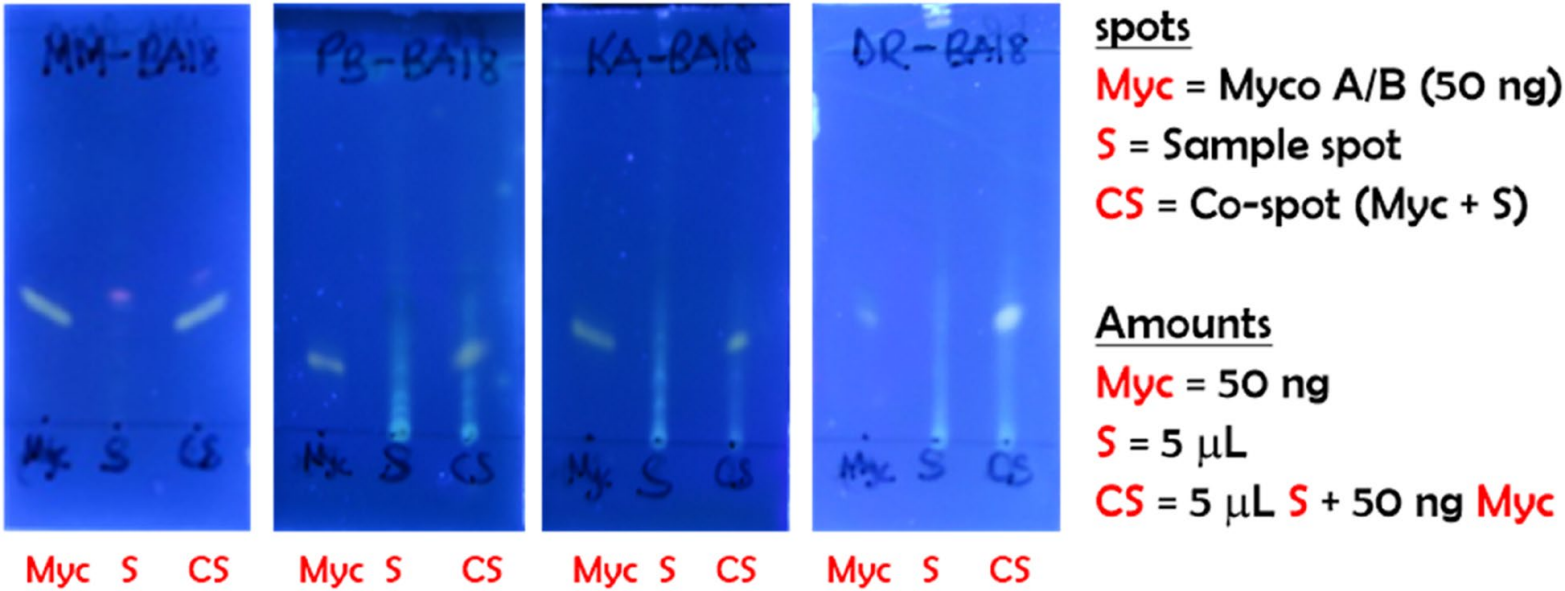
f-TLC plates of clinical samples from 4 different PCR negative confirmed non-BU cases. Each TLC plate is marked with three spots, Myco, S and CS for synthetic mycolactone A/B, sample, and co-spot respectively. The samples were applied at the same concentration and the plates were developed using chloroform/hexane/methanol (5:4:1; v/v/v)

A comparative study between the promising boronic acid (BA18) and the original boronic acid (BA) using the f-TLC method was conducted on five (5) different PCR positive confirmed BU patients. Two sets of measurements were conducted (Fig. 10), first, the original BA (top panel) was used to form the adducts according to previously described protocols followed by B18 (bottom panel). Like the results obtained with non-clinical samples, B18 showed an improved intensity of the myco-B18 adduct spot versus that with BA. In addition, it was easier to interpret the TLC profiles of the samples with background interference exemplified by unique identifiers SA and LN respectively. Also, four (4) different PCR negative control non-BU samples were analyzed using our most promising BA18 boronic acid to allow for comparison with the positive cases (Fig. 11). Here, it is worthy of note that despite the background interferences resulting from the co-extracted human tissue lipids from the samples, the TLC profiles clearly indicated negative results because the BA18 is strongly fluorescent as compared to the original BA as presented on the top panel of Fig. 10.

## Discussion

Prior to direct observation under a 365 nm UV light, fluorescent complexation reactions of various commercially available arylboronic acids with the 1,3-diol moieties on the mycolactone A/B structure on a TLC surface were carried out. Mycolactone A/B is weakly fluorescent in its free form; however, it becomes fluorescent when bound to an appropriate boronic acid. BA has been the boronic acid of choice for the f-TLC method [19].

Woodward-Fieser rule has been employed in correlating electronic transitions and structures of α,β-unsaturated ketones as well as forecasting the position of the longest wavelength π → π* band of these compounds [47, 48]. Here, we used the Woodward-Fieser rule to calculate a predicted empirical wavelength for the lowest energy π → π* electronic transition of mycolactone. For α,β-unsaturated esters, the base value is 215 nm. A total of 4 additional double bonds from the pentaenoate region (+ 30 each), and 3 methyl-substituted double bonds contributing + 18 each were added. The sum of the calculated absorption maximum was 367 nm compared to the experimental value of 362 nm as measured on UV. This is consistent with the UV–vis analysis of the fatty acid side chain of mycolactone A/B [44]. These findings demonstrate that even though 2 cyclic boronates are formed, one on the southern side chain diol and the other on the northern side chain, only the boronate of the southern side chain with the chromophore was excited by irradiation through a 365 nm filter. The boronate on the upper chain, however, was not excited and hence did not contribute to the enhanced fluorescence under UV light [49]. The arylboronate formed has the potential to enhance fluorescence emission [44, 45]. This is because the excitation of mycolactone alone presented weak fluorescence at 537 nm with a Stokes shift of 175 nm (Fig. 7A). However, after the complexation was triggered with the boronic acid, a significant turn-on fluorescent enhancement was observed at the same 537 nm, and the fluorescence was also visually observed on TLC using a UV lamp, as shown in Fig. 7B. Thus, the change in fluorescence intensity of mycolactone A/B is attributed to an energy transfer from the excited pentaenoate motif which serves as a fluorophore to a fluorogenic acceptor in the form of the arylboronic acid (BA18) [19].

## Conclusions

Twenty-six commercially available arylboronic acids were screened as potential alternatives for BA. TLC analyses of the purchased boronic acids were performed and their fluorescence band intensities profiles were compared to the original BA. The boronic acids readily form arylboronate ester complexes with mycolactone A/B and three of them gave superior fluorescence band intensities. A repeat of the complexation of the boronic acids BA, BA15, BA18, and BA21 in serially diluted BA from 10 ng/µL – 90 ng/µL gave similar results with BA18 emerging as the most visibly intense fluorescence bands.

When UV–vis measurements were performed with mycolactone A/B and myco-boronic acid adducts. Absorption maxima (λ_max_) of 362 nm for mycolactone A/B was obtained while that of BA15, BA18, and BA21 were 272 nm, 270 nm, and 286 nm respectively. Applying the Woodward-Fieser rule, only the boronate of the southern side chain with the chromophore was excited by irradiation through a 365 nm filter while that of the northern side was not.

The fluorescence intensity of the boronate ester formation between mycolactone A/B and boronic acids increased as the concentration of mycolactone increased and the formation of the myco-BA15 boronate ester adduct was confirmed by HR-MS. Finally, a comparative study between BA18 and BA using PCR confirmed patient samples gave an improved intensity of the adduct and an easier to interpret TLC profiles of the samples even for those with background interference.

## Supplementary Information

**Additional file 1: Table S1.** List of attempted boronic acids with their corresponding TLCs. **Figure S1.** ESI mass spectrum of mycolactone A/B. **Figure S2.** ESI mass spectrum of mycolactone A/B–BA15 boronate adduct.

## Data Availability

All data generated or analyzed during this study are included in this published article and its supplementary information files.

## Abbreviations

f-TLC: Fluorescent-thin layer chromatography
TLC: Thin layer chromatography
BU: Buruli ulcer
Non-BU: Non-Buruli ulcer
HR-MS: High Resolution Mass Spectrometry
MU: *Mycobacterium ulcerans*
PCR: Polymerase Chain Reaction
UV: Ultraviolet
LAMP: Loop-mediated isothermal amplification
RNA: Ribonucleic acid
WHO: World Health Organization
Q-ToF: Quadrupole-Time of Flight tandem mass spectrometer
