# Supplementary material for: Alternative boronic acids in the detection of Mycolactone A/B using the thin layer chromatography (f-TLC) method for diagnosis of Buruli ulcer
Source: BMC Infect Dis. 2023 Jul 27;23:495. doi: 10.1186/s12879-023-08426-2 (PMC10373253; doi:10.1186/s12879-023-08426-2)
Supplement: Supplementary file 1 [file 12879_2023_8426_MOESM1_ESM.odt]

Table S1: List of attempted boronic acids with their corresponding TLCs

| Entry | Boronic acid | Structure | TLC |
| --- | --- | --- | --- |
| BA | 2-naphthylboronic acid |  |  |
| BA1 | Pyrene-1-boronic acid |  |  |
| BA2 | 4-Phenyl(napthalene-1-yl)boronic acid |  |  |
| BA3 | 4-Methyl-1-naphthaleneboronic acid |  |  |
| BA4 | 6-Ethoxy-2-naphthaleneboronic acid |  |  |
| BA5 | 9-phenanthreneboronic acid |  |  |
| BA6 | 4-(Naphthalen-2-yl)phenylboronic acid |  |  |
| BA7 | Dibenzo[b,d]thiophen-2-ylboronic acid |  |  |
| BA8 | (4-Bromonaphthalen-1-yl)boronic acid |  |  |
| BA9 | 4-aminomethylphenylboronic acid, hydrochloride |  |  |
| BA10 | 4-(Bromomethyl)phenylboronic acid |  |  |
| BA11 | 4-Formylphenylboronic acid |  |  |
| BA12 | (4'-bromo-[1,1'-biphenyl]-4-yl)boronic acid |  |  |
| BA13 | (4-(9-Phenyl-9H-carbazol-3-yl)phenyl)boronic acid |  |  |
| BA14 | 10-(2-Naphthyl)anthracene-9-boronic acid |  |  |
| BA15 | [1,1':4',1''-terphenyl]-2-ylboronic acid |  |  |
| BA16 | 4-Hydroxylphenylboronic acid |  |  |
| BA17 | 4-(4-Biphenyl)phenyl boronic acid |  |  |
| BA18 | (9,9-Diphenyl-9H-fluoren-4-yl)boronic acid |  |  |
| BA19 | [1,1'-Binaphthalen]-4-ylboronic acid |  |  |
| BA20 | (4'-Ethoxy-[1,1'-biphenyl]-4-yl)boronic acid |  |  |
| BA21 | (3-(Naphthalen-1-yl)phenyl)boronic acid |  |  |
| BA22 | 3-(2-Naphthyl)phenylboronic acid |  |  |
| BA23 | 2-Biphenylboronic acid |  |  |
| BA24 | 3-Biphenylboronic acid |  |  |
| BA25 | 9,9-Dimethyl-9H-fluoren-2-ylboronic acid |  |  |

Text

Description automatically generated with low confidence

Figure S1: ESI mass spectrum of mycolactone A/B. 

Diagram

Description automatically generated

Figure S2: ESI mass spectrum of mycolactone A/B–BA15 boronate adduct.
